# The Single-Visit Approach as a Cervical Cancer Prevention Strategy Among Women With HIV in Ethiopia: Successes and Lessons Learned

**DOI:** 10.9745/GHSP-D-15-00325

**Published:** 2016-03-25

**Authors:** Netsanet Shiferaw, Graciela Salvador-Davila, Konjit Kassahun, Mohamad I Brooks, Teklu Weldegebreal, Yewondwossen Tilahun, Habtamu Zerihun, Tariku Nigatu, Kidest Lulu, Ismael Ahmed, Paul D Blumenthal, Mengistu Asnake

**Affiliations:** a Pathfinder International, Addis Ababa, Ethiopia; b Pathfinder International, Watertown, MA. Now independent consultant.; c Pathfinder International, Watertown, MA, and Boston University School of Public Health, Boston, MA, USA; d Centers for Disease Control and Prevention, Addis Ababa, Ethiopia; e Stanford Program for International Reproductive Education and Services (SPIRES), Stanford University School of Medicine, Stanford, CA, USA

## Abstract

**Introduction:**

Cervical cancer is the second most common form of cancer for women in Ethiopia. Using a single-visit approach to prevent cervical cancer, the *Addis Tesfa* (New Hope) project in Ethiopia tested women with HIV through visual inspection of the cervix with acetic acid wash (VIA) and, if tests results were positive, offered immediate cryotherapy of the precancerous lesion or referral for loop electrosurgical excision procedure (LEEP). The objective of this article is to review screening and treatment outcomes over nearly 4 years of project implementation and to identify lessons learned to improve cervical cancer prevention programs in Ethiopia and other resource-constrained settings.

**Methods:**

We analyzed aggregate client data from August 2010 to March 2014 to obtain the number of women with HIV who were counseled, screened, and treated, as well as the number of annual follow-up visits made, from the 14 tertiary- and secondary-level health facilities implementing the single-visit approach. A health facility assessment (HFA) was also implemented from August to December 2013 to examine the effects of the single-visit approach on client flow, staff workload, and facility infrastructure 3 years after initiating the approach.

**Results:**

Almost all (99%) of the 16,632 women with HIV counseled about the single-visit approach were screened with VIA during the study period; 1,656 (10%) of them tested VIA positive (VIA+) for precancerous lesions. Among those who tested VIA+ and were thus eligible for cryotherapy, 1,481 (97%) received cryotherapy treatment, but only 80 (63%) women eligible for LEEP actually received the treatment. The HFA results showed frequent staff turnover, some shortage of essential supplies, and rooms that were judged by providers to be too small for delivery of cervical cancer prevention services.

**Conclusion:**

The high proportions of VIA screening and cryotherapy treatment in the *Addis Tesfa* project suggest high acceptance of such services by women with HIV and feasibility of implementation in secondary- and tertiary-level health facilities. However, success of cervical cancer prevention programming must address wider health system challenges to ensure sustainability and appropriate scale-up to the general population of Ethiopia and other resource-constrained settings.

## Abstract (precis)

With the single-visit approach for cervical cancer prevention, women with positive “visual inspection of the cervix with acetic acid wash” (VIA) test results receive immediate treatment of the precancerous lesion with cryotherapy. The approach worked successfully for women with HIV in Ethiopia in secondary and tertiary health facilities, with high screening and cryotherapy treatment rates. Sustainability and appropriate scale-up of such programs must address wider health system challenges including human resource constraints and shortage of essential supplies.

## INTRODUCTION

Cancer of the uterine cervix is an important public health challenge in low- and middle-income countries (LMICs). In 2012, almost 270,000 women died from cervical cancer, 86% of them in less developed regions of the world.^1^ The majority of women who die from cervical cancer in LMICs are in the prime of their life, resulting in social and economic repercussions for both their families and their communities.^2^ With early detection and effective management, cervical cancer is one of the most preventable and treatable forms of cancer.^3^ Unfortunately, the majority of women with cervical cancer in LMICs are diagnosed at late stages of the disease and do not have access to lifesaving treatment or prevention options.^3^ A series of seminal studies have proven the safety, acceptability, and effectiveness of the single-visit approach for cervical cancer prevention in low-income countries.^4^^–^^7^ With the single-visit approach, women are tested through visual inspection of the cervix with acetic acid wash (VIA) and, if they test positive, receive immediate treatment of the precancerous lesion with cryotherapy, a procedure that freezes and destroys diseased tissue.

With early detection and effective management, cervical cancer is one of the most preventable and treatable forms of cancer.

Screening is considered optimal when the smallest amounts of resources are used to achieve the greatest benefit. Cervical cancer screening with VIA is a simple and affordable alternative to cytology-based screening with accuracy to detect precancerous lesions at a rate comparable with or better than cytology.^8^^,^^9^ Furthermore, nurses, midwives, and other non-physician health care providers can be trained in VIA and cryotherapy, which can greatly improve access to cervical cancer prevention services.^10^^–^^12^ In contrast, the sensitivity rate of HPV DNA testing (94%) is much higher than VIA (80%)^9^^,^^13^; however, the high cost and health system requirements of HPV DNA testing would be challenging for large-scale implementation in LMICs. The addition of loop electrosurgical excision procedure (LEEP) services offers women with positive VIA results and larger lesions a good alternative to hysterectomy, the only previously available treatment option in Ethiopia and most other LMICs. With the “global call to action” for cancer control in low-income countries,^14^ various multi-stakeholder cervical cancer prevention initiatives have been implemented in LMICs.

Cervical cancer screening with visual inspection of the cervix with acetic acid wash (VIA) is simple and affordable.

This article describes the *Addis Tesfa* (New Hope) project, the first cervical cancer prevention program in Ethiopia that uses the single-visit approach. Ethiopia is a low-income country located in the horn of Africa. In 2012, the country had a gross domestic product (GDP) per capita of US$472 and spent 3.8% of GDP on total health expenditure—about half (48%) of this expenditure was made by the public sector.^15^ Lower respiratory infections, cancer, diarrheal diseases, malaria, tuberculosis, and HIV remain important public health challenges in the country. In 2012, cervical cancer in Ethiopia was reported to be the second leading cancer diagnosis (after breast cancer) among adult women with an estimated 7,095 new cases and 4,732 deaths.^1^ Routine access to cervical cancer screening was not available and treatment for precancerous cervical lesions did not exist in Ethiopia until implementation of the *Addis Tesfa* project in 2009. The objective of this paper is to review screening and treatment outcomes of the *Addis Tesfa* project from August 2010 through March 2014 using routine monitoring data and to identify lessons learned from this project in order to increase access to cervical cancer prevention programs in Ethiopia and other resource-constrained settings.

## METHODOLOGY

### Context and Project Sites

Funded by the US Centers for Disease Control and Prevention (CDC), Pathfinder International’s *Addis Tesfa* (New Hope) project was launched in 2009, in collaboration with the Ethiopian Federal Ministry of Health (FMOH) and the Stanford University Program for International Reproductive Education and Services, to introduce cervical cancer prevention services in Ethiopia for women with HIV. Although the Ethiopian adult HIV prevalence of 1.3% is much lower than the sub-Saharan African regional average of 4.7%,^16^ the rationale to offer the single-visit approach to women with HIV was based on global evidence that such women have higher rates of cervical dysplasia and lesions than women without HIV.^17^^–^^20^ In addition, targeting women with HIV was an ideal entry point to assess the single-visit approach in Ethiopia prior to national scale-up and integration into routine health services.

Fourteen sites, which represented tertiary- and secondary-level health facilities in Ethiopia, were selected based on client volume, access to HIV services, geographic location, and readiness of the Regional Health Bureaus and hospital managers to participate. The 14 sites were located in 4 of the 9 geographical regions of Ethiopia in addition to the administrative capital, Addis Ababa. One facility, often a regional referral center or a university-based teaching hospital, was selected to be a Center of Excellence in each of the selected regions to provide cervical cancer prevention training; later these centers offered LEEP treatment as an alternative for women ineligible for cryotherapy. The majority of women with HIV attending the HIV/AIDS care and treatment service units at these 14 sites were on antiretroviral therapy (ART).

The *Addis Tesfa* project, launched in 2009, introduced cervical cancer prevention services in 14 sites in Ethiopia.

### Provider Training

A total of 77 health care providers (51 nurses/midwives and 26 physicians) were trained for VIA and cryotherapy in the selected sites from July 2010 to July 2013. Each site trained teams of 1–2 physicians and 3–5 nurses/midwives to provide cervical cancer prevention services through the single-visit approach. Trained physicians working in the 5 Centers of Excellence and who were certified in VIA and cryotherapy were also trained on LEEP.

To facilitate the training, the *Addis Tesfa* project developed basic clinical and counseling guides, client consent forms, SVA standard operating procedures, and quality management toolkits (http://www.pathfinder.org/publications-tools/cervical-cancer-prevention.html). The competency-based training program combined didactic sessions with practical skill development modules; the training programs lasted 10 days for nurses/midwives and 5 days for obstetricians and gynecologists.

To ensure clinical competence, semiannual refresher trainings and quarterly quality control visits were conducted jointly with several Regional Health Bureau staff members. During these visits, supervisors used checklists developed by the project to verify health care providers’ skills in interpersonal communication and counseling, VIA, cryotherapy, LEEP, and adherence to infection prevention guidelines.

### Single-Visit Approach

The clinical protocol for the single-visit approach was based on World Health Organization guidelines for screening and treatment of precancerous lesions for cervical cancer prevention^21^ but was modified and adapted to the Ethiopian context through expert consultation. Women with HIV aged 30–45 years received pre-procedure counseling on cervical cancer screening and treatment; written client consent was required prior to treatment of precancerous lesions. Women were screened using VIA and categorized as VIA negative (VIA-), VIA positive (VIA+), VIA inconclusive, or suspected of having cancer based on visual examination. All results were carefully explained to the women and documented on facility records.

The Addis Tesfa project, launched in 2009, introduced cervical cancer prevention services in 14 sites in Ethiopia.

Women with VIA- test results were counseled to come back for subsequent screening 5 years later. Women with VIA+ results who were eligible for cryotherapy were offered immediate treatment and counseled to return 1 year later for follow-up screening. Those not eligible for cryotherapy due to large lesion size (precancerous lesion >75% of cervix and lesion extending >2 mm beyond the diameter of the cryosurgical tip) were referred for LEEP at one of the 5 Centers of Excellence and also counseled to return in 1 year for follow-up screening (Box ). Women with suspected cancer through visual examination or with inconclusive VIA screening results were referred for further diagnosis and management.

**BOX. Cryotherapy and LEEP Procedures Employed by the Addis Tesfa Project in Ethiopia**

**Cryotherapy**

- A standard double-freeze cryotherapy technique was employed (freeze for 3 minutes, thaw for 5 minutes, freeze for another 3 minutes) in order to create an ice-ball that extended 4–5 mm beyond the cryotherapy tip edge.
- Post-cryotherapy counseling was given, which included self-care and guidance for any potential complications. Women were counseled for subsequent screening 1 year later, which was documented on the client card with the rescreening date highlighted.

**Loop Electrosurgical Excision Procedure (LEEP)**

- LEEP excisional biopsy and conization (using a loop or triangular electrode to excise a cervical cone) are procedures performed in an outpatient setting under local anesthesia.
- LEEP was postponed for 4 weeks if cervicitis was present; antibiotics were prescribed based on national guidelines for women with HIV.
- Post-LEEP counseling was given, which included self-care and guidance for any adverse effects. Women were counseled for subsequent screening at 6 weeks and at 1 year.

### Health Facility and Community Demand Creation

The project invested early efforts in educating health professionals, policy makers, and local leaders about cervical cancer prevention. The project held a series of sensitization and orientation workshops to build key partnerships and to garner support for services, and to ultimately promote the sustainability of services. To create awareness of the new cervical cancer prevention services, the project developed information, education, and communication materials tailored for the Ethiopian context. Information on cervical cancer prevention was disseminated at health facilities to people living with HIV/AIDS (PLHIV). In addition, sensitization and orientation to cervical cancer prevention services were provided to health professionals, policy makers, and local HIV associations through group workshops. Community awareness was also delivered through various media channels including radio, newspaper, and television announcements starting in November 2011.

### Timeline of Project Rollout

The *Addis Tesfa* project began offering cervical cancer prevention services to women with HIV in the 5 regional Centers of Excellence in August 2010. An additional 4 sites provided the single-visit approach to cervical cancer prevention by September 2011, and the remaining 5 sites began providing the services by August 2012 (14 sites total).

### Data Collection and Analysis

Screening and treatment services were documented in facility records. Each facility aggregated counseling, screening, and treatment results from the single-visit approach into monthly summary forms, which were entered into an electronic database for analysis by the *Addis Tesfa* project. Routine monitoring data that were collected from all 14 sites included the number of women with HIV who:

- Were counseled about the single-visit approach
- Were screened with VIA (stratified by VIA screening results)
- Tested VIA+ and who received treatment
- Tested VIA+ and who received treatment and returned for follow-up

Quality control of monitoring data was conducted through monthly data checks by health facility and *Addis Tesfa*’s regional project staff members at each site, augmented by additional data review by the project data manager once monthly data were submitted to the *Addis Tesfa* project.

Basic descriptive statistics and trend analysis were conducted retrospectively on project data from August 2010 to March 2014 using SPSS v. 20. In addition, global chi-square test of independence at α = .05 significance level was performed to assess regional variability on key single-visit approach results.

A health facility assessment (HFA) was conducted by Pathfinder International and respective hospital management teams from August to December 2013 to assess the effects of introduction of the single-visit approach on the health systems of individual facilities. To examine the site’s client flow, staff workload, and facility infrastructure, equipment, and supplies 3 years after initiation of the single-visit approach, an HFA tool was developed for this project that recorded observations and staff feedback at all 14 single-visit approach sites.

### Ethical Considerations

In addition to ensuring clinical competence, health care provider training and refresher courses reinforced the importance of client consent and privacy during service provision. Ethical clearance was obtained from the US Centers for Disease Control and Prevention (CDC) who determined that data collection and analysis conducted as part of this programmatic review was not considered human subjects research.

## RESULTS

### Screening and Treatment Coverage

**FIGURE 1.**
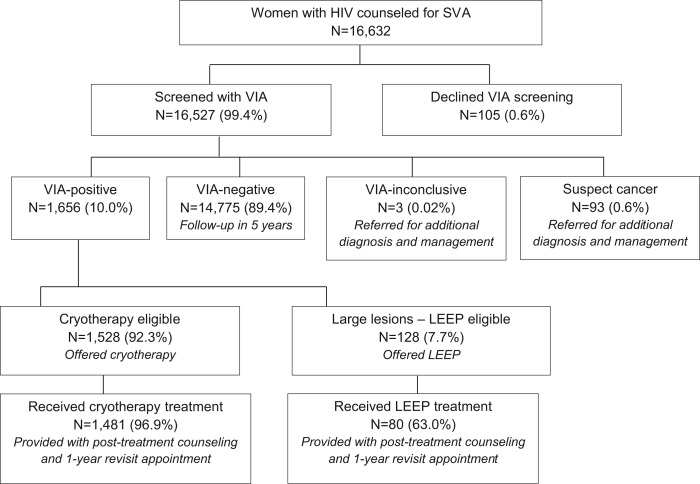
Single-Visit Approach Flow Chart for Cervical Cancer Prevention Services Provided to Women With HIV, August 2010 to March 2014 Abbreviations: LEEP, loop electrosurgical excision procedure; SVA, single-visit approach; VIA, visual inspection of the cervix with acetic acid wash.

**FIGURE 2.**
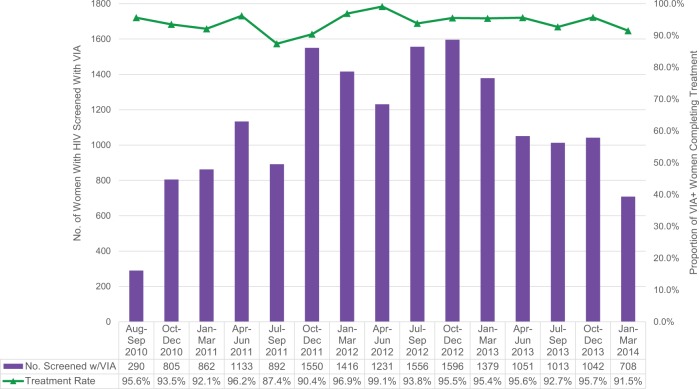
Number of Women With HIV Screened With VIA and Treatment Rate With Cryotherapy or LEEP Across All Project Sites, August 2010 to March 2014 Abbreviations: LEEP, loop electrosurgical excision procedure; VIA, visual inspection of the cervix with acetic acid wash.

Between August 2010 and March 2014, the project counseled 16,632 women with HIV about the single-visit approach (Figure 1). Nearly all of these women (99.4%) agreed to be screened with VIA, and of those screened, 10.0% (n = 1,656) had positive VIA test results (Figure 1). The number of women with HIV screened with VIA increased from 290 in the first period of service delivery (August–September 2010) to 1,596 in the tenth quarter (October–December 2012) as the number of facilities providing the single-visit approach services increased, but decreased to 708 in the final quarter (January–March 2014) of this project as a result of the declining pool of eligible women with HIV seeking the services (Figure 2). The overall treatment rate—the percentage of women with HIV who had positive VIA test results and were treated with either cryotherapy or LEEP—averaged 94.3% from August 2010 to March 2014, and it remained at this relatively high proportion throughout the project period (range, 87.4% to 99.1%).

More than 16,000 women with HIV were counseled about the single-visit approach, and nearly all agreed to be screened with VIA.

The overall treatment rate among women with VIA+ test results averaged 94% during the project period.

### Initial VIA Screening by Region

There was regional variability in the results of the initial VIA screening (χ^2^ = 103.4; df = 8; *P*<.001). In regards to VIA positivity, the Southern Nations, Nationalities, and People's Region (SNNPR) showed the highest rates of precancerous lesions (13.1%) while Amhara had the lowest rate (7.3%) (Table 1). There were also significant differences between regions in eligibility for cryotherapy and LEEP (χ^2^ = 25.5; df = 4; *P*<.001). Among women who tested VIA+, 1,529 (92.3%) women with HIV were eligible for cryotherapy (range, 88.6% in Addis Ababa to 97.3% in Oromia). Only 128 (7.7%) women with HIV who tested VIA+ were referred for LEEP (range, 2.7% in Oromia to 11.4% in Addis Ababa). Few women with HIV (n = 93, or 0.6%) were suspected to have cancer based on visual inspection and were referred for additional testing and clinical management (range, 0.1% in Tigray to 0.8% in Amhara).

**TABLE 1:** Results of VIA Screening in Ethiopia by Region, August 2010 to March 2014

	Addis Ababa (2 sites)	Amhara (3 sites)	Oromia (3 sites)	SNNPR (3 sites)	Tigray (3 sites)	Total (14 sites)
Screening^a^	n = 2,066	n = 5,688	n = 2,574	n = 2,619	n = 3,577	n = 16,524
VIA+	245 (11.9%)	416 (7.3%)	258 (10.0%)	343 (13.1%)	394 (11.0%)	1,656 (10.0%)
VIA−	1,806 (87.4%)	5,229 (91.9%)	2,303 (89.5%)	2,258 (86.2%)	3,179 (88.9%)	14,775 (89.4%)
Suspect cancer	15 (0.7%)	43 (0.8%)	13 (0.5%)	18 (0.7%)	4 (0.1%)	93 (0.6%)
Eligibility among VIA+	n = 245	n = 416	n = 258	n = 343	n = 394	n = 1,656
Cryotherapy eligible	217 (88.6%)	395 (95.0%)	251 (97.3%)	316 (92.1%)	349 (88.8%)	1,528 (92.3%)
LEEP eligible	28 (11.4%)	21 (5.0%)	7 (2.7%)	27 (7.9%)	45 (11.2%)	128 (7.7%)

Abbreviations: LEEP, loop electrosurgical excision procedure; SNNPR, Southern Nations, Nationalities, and People's Region; VIA, visual inspection of the cervix with acetic acid wash.

a Three inconclusive VIA test results are excluded from this table.

### Treatment Acceptance

Among women with HIV who tested VIA+ and were eligible for cryotherapy during the life of the project, 1,481 (96.9%) received cryotherapy treatment with almost all (98%) women receiving treatment on the same day of screening (Table 2). There were regional differences with VIA+ women who received cryotherapy (χ^2^ = 43.6; df = 4; *P*<.001). The proportion of women treated with cryotherapy was as low as 93.1% (Addis Ababa) to as high as 100% (Tigray). Although regional differences were detected, the great majority of women who were eligible received cryotherapy throughout the different regions.

**TABLE 2:** Treatment Results of the Single-Visit Approach in Ethiopia by Region, August 2010 to March 2014

	Addis Ababa (2 sites)	Amhara (3 sites)	Oromia (3 sites)	SNNPR (3 sites)	Tigray (3 sites)	Total (14 sites)
Cryotherapy eligible	n = 217	n = 395	n = 251	n = 316	n = 349	n = 1,528
Received cryotherapy	202 (93.1%)	394 (99.7%)	236 (94.0%)	300 (94.9%)	349 (100.0%)	1,481 (96.9%)
Did not receive cryotherapy	15 (6.9%)	1 (0.3%)	15 (6.0%)	16 (5.1%)	0 (0.0%)	47 (3.1%)
LEEP eligible	n = 28	n = 21	n = 7	n = 27	n = 45	n = 128
Received LEEP	22 (78.6%)	1 (4.8%)	3 (42.9%)	15 (55.6%)	39 (86.7%)	80 (62.5%)
Did not receive LEEP	6 (21.4%)	20 (95.2%)	4 (57.1%)	12 (44.4%)	6 (13.3%)	48 (37.5%)

Abbreviations: LEEP, loop electrosurgical excision procedure; SNNPR, Southern Nations, Nationalities, and People's Region.

The percentage of women who were referred for LEEP and received treatment was considerably lower than for cryotherapy; only 80 (63.0%) women eligible for LEEP actually received the treatment. Regional variability was observed in the percentage of women with HIV receiving LEEP treatment (χ^2^ = 45.9; df = 4; *P*<.001), from only 1 client (4.8%) receiving LEEP in Amhara to 39 clients (86.7%) in Tigray.

The treatment rate for LEEP (63%) was considerably lower than for cryotherapy (97%).

### Follow-Up Screening

Women with HIV who were VIA+ and received treatment with cryotherapy or LEEP were expected to be rescreened 1 year later. From August 2010 to March 2014, about half (51.1%) of the 1,201 women expected to come for follow-up actually returned for screening 1 year later and were screened (Table 3).

**TABLE 3:** One-Year Follow-Up Results of the Single-Visit Approach in Ethiopia by Region, August 2010 to March 2014

	Addis Ababa (2 sites)	Amhara (3 sites)	Oromia (3 sites)	SNNPR (3 sites)	Tigray (3 sites)	Total (14 sites)
Women expected for the rescreening	n = 178	n = 277	n = 188	n = 272	n = 286	n = 1,201
Returned for rescreening	53 (29.8%)	153 (55.2%)	71 (37.8%)	105 (38.6%)	232 (81.1%)	614 (51.1%)
Did not return for rescreening	125 (70.2%)	124 (44.8%)	117 (62.2%)	167 (61.4%)	54 (18.9%)	587 (48.9%)
Follow-up screening test results	n = 52	n = 153	n = 71	n = 105	n = 232	n = 613
VIA	40 (76.9%)	143 (93.5%)	63 (88.7%)	96 (91.4%)	208 (89.7%)	550 (89.6%)
VIA+	12 (23.1%)	10 (6.5%)	7 (9.9%)	9 (8.6%)	24 (10.3%)	62 (10.1%)
Suspect cancer	0 (0.0%)	0 (0.0%)	1 (1.4%)	0 (0.0%)	0 (0.0%)	1 (0.2%)

Abbreviations: SNNPR, Southern Nations, Nationalities, and People's Region; VIA, visual inspection of the cervix with acetic acid wash.

One-year follow-up rates differed greatly between regions (χ^2^ = 167.8; df = 4; *P*<.001); the proportion of women returning for screening was as low as 29.8% in Addis Ababa to as high as 81.1% in Tigray. Results from the 1-year follow-up VIA rescreening indicated that 90% of women with HIV who were VIA+ 1 year prior were VIA- at rescreening and potentially cured from the precancerous lesion that was detected from the original VIA screening; 0.2% of women were suspected to have cancer. Differences in the 1-year follow-up VIA screening results were detected between the various regions (χ^2^ = 19.7; df = 8; *P* = .01). The 2-year follow-up rates were much lower with only 36.8% (14 of 38) of women with HIV expected to be rescreened actually returning for the single-visit approach services; however, screening results among the 14 women who returned at 2 years indicated they were all VIA-.

About half of the women counseled to return for rescreening 1 year later actually returned.

### Health Facility Assessment

The results from the HFA can be found in Table 4. Interruption in clean water supply and electricity was detected in the majority of sites. Although all sites provided private rooms for the single-visit approach, a small number of facilities was judged by providers to have rooms that were not wide enough for cervical cancer prevention equipment to comfortably conduct patient counseling, screening, treatment, and infection prevention procedures. In addition, HFA findings indicated that a number of facilities faced difficulties with their cryotherapy units, especially with the freezing and de-freezing components of the instruments. Shortage of supplies such as speculums, forceps, and hemostatic agents (Monsel’s solution) was occasionally experienced at some sites. Finally, staff turnover was identified as an important challenge; only three-quarters of health workers trained in the single-visit approach were still working at their respective sites and 4 sites did not have a gynecologist trained in the single-visit approach during the HFA. Among health workers providing the single-visit approach services, there were complaints associated with their work load as they were assigned to multiple services, such as coverage for the single-visit approach and HIV services during the same shift.

Several health system challenges were identified including inconsistent water and electricity supply and staff turnover.

**TABLE 4:** Key Single-Visit Approach Findings From the Health Facility Assessment, Ethiopia, 2013

Health System Areas	Key Observations
**Infrastructure**	
Electricity	Interruption in power supply was noted at all 14 sites.
Back-up generator	All 14 sites had back-up generators; however, 1 site did not have back-up system connected to the CCP room.
Water supply	10 of 14 sites noted frequent interruption to water supply.
Examination rooms	All 14 sites had a private room for CCP services; however, about one-quarter (4 of 14) of the sites had rooms that were of insufficient size.
**Equipment and Supplies**	
Cryo-machine	Over one-quarter (4 of 14) of the sites reported a problem with the cryo-machine.
Spare parts	Majority of sites (12 of 14) had an extra O-ring available.
CCP supplies	About three-quarters (10 of 14) of the sites had all the necessary CCP supplies available with no shortages detected.
Other equipment	A few sites (2 of 14) noted that the examination lamp was not functional and required maintenance.
**Staff**	
Retention	Three-quarters (57 of 77) of health workers that were trained in SVA were still working at the same site.
Workload	Majority of SVA providers at all 14 sites complained of the workload as they were also assigned to a different service unit on the same working day.

Abbreviations: CCP, cervical cancer prevention; SVA, single-visit approach.

## DISCUSSION

Results from the *Addis Tesfa* single-visit approach project are consistent with published literature of cervical cancer prevention programs using VIA and treatment with cryotherapy and LEEP in low-resource settings.^5^^,^^6^^,^^10^^,^^22^^–^^24^ The increasing trend in the total number of women with HIV screened with VIA from 2010 to 2012 was associated with the increasing number of sites providing the single-visit approach (all 14 selected sites were providing such services by August 2012) and the ongoing community awareness and demand creation activities. The decline in the total number of women with HIV screened with VIA from 2012 to 2014 can most likely be attributed to the decreasing pool of eligible women with HIV seeking single-visit approach services in the 14 project-supported sites. Programmatic data suggest that approximately 85% to 90% of eligible women with HIV in the project catchment area received the single-visit approach service.^25^

The project’s overall VIA+ rate of 10.0% is relatively low compared with other studies using VIA for women with HIV; for example, the VIA+ rate among women with HIV in Guyana^24^ and Tanzania^26^ was 16% and 27%, respectively. The reason for the low VIA+ prevalence in this project could be partially attributed to the fact that the women in our study may not have been severely immunocompromised. Women in the *Addis Tesfa* project were recruited from HIV care and treatment centers in secondary and tertiary health facilities and may have already been taking ART, which could have improved their immune system. Unfortunately, this study was not designed to capture individual women's characteristics associated with HIV status and treatment history. Anecdotal data indicate that approximately three-fourths of women with HIV in the study population were on ART.^25^ Another possible explanation for the low VIA+ rate may be due to under-diagnosis by service providers. Providers at all project-supported facilities received competency-based training, which was complemented by routine self-learning using different educational materials (training materials, flash cards, educational videos, etc.) and onsite mentoring by gynecologists. In addition, all single-visit approach service providers were given refresher trainings and regular mentoring visits by *Addis Tesfa* project personnel. However, despite the project’s quality improvement measures, it is possible that variability in the skills and competency of service providers could have resulted in under-diagnosis that led to the project’s low prevalence of VIA+.

The relatively low VIA+ rate of 10% in the project setting may be because most women were on ART and thus may not have been immunocompromised.

The project’s overall cryotherapy treatment rate was high with 96.9% of women eligible for cryotherapy receiving treatment. In other single-visit approach studies, the cryotherapy treatment rate was 94% in Guyana,^24^ 92% in Thailand,^6^ 91% in Ghana,^5^ 84% in Laos,^22^ and 61% in the demonstration project in Malawi, Madagascar, Nigeria, Tanzania, Uganda, and Zambia.^23^ Our project’s high cryotherapy rate is most likely a result of this procedure being truly offered in a single visit. In addition, the rights-based approach to training health care providers that emphasized comprehensive client counseling, respect for confidentiality and privacy, and informed consent may also have facilitated the high cryotherapy rate.

The low LEEP eligibility rate of 7.7% is most likely due to the late introduction of LEEP, which was introduced into the *Addis Tesfa* project starting in November 2011—15 months after the start of the project, in the 5 Centers of Excellence. As LEEP was only offered in these 5 centers, the low LEEP treatment rate of 63% is most likely a result of women who were referred for LEEP but were unable or unwilling to travel to one of the Centers of Excellence to obtain LEEP services. To reach a Center of Excellence health facility, a client may need to travel 40–500 kilometers and spend anywhere from US$5–50 for round-trip transportation costs. Cervical cancer prevention services that require additional procedures or services at other sites are no longer “single-visit approaches” and will inevitably reduce overall treatment rates. LEEP treatment rates for clients at Centers of Excellence were relatively high (>78%); however, challenges were observed in some project-supported health facilities. In the Amhara center, the 1-year delay in the installation of the LEEP machine could have resulted in lower treatment rates in this region. In addition, the Center of Excellence in Oromia region did not offer LEEP regularly as there was high turnover of a LEEP-trained gynecologist in the region, contributing to lower referral and treatment rates for LEEP.

A little over half (51%) of women with HIV in the *Addis Tesfa* project returned for follow-up and rescreening 1 year later. In other single-visit approach programs in low-resource settings, 1-year follow-up rates were 53% in Ghana,^5^ 50% in Guyana,^24^ and 50% in the demonstration project in 6 African countries.^23^ The Ethiopian experience in regards to 1-year follow-up for the single-visit approach is almost identical to the findings in Guyana and in the African countries. This is in contrast to the Southeast Asian demonstration studies, which provided single-visit approach services in rural settings through primary health centers. These demonstration studies showed impressively high 1-year follow-up rates of 94% in Thailand^6^ and 68% in Laos.^22^ The 2-year follow-up rates showed that approximately one-third (37%) of women expected to be rescreened actually returned for follow-up, highlighting the challenges of long-term follow-up in this program. It can be assumed that the proportion of follow-up and rescreening in the subsequent years will continue to decline. To ensure the effectiveness of the single-visit approach, programs need to develop systems and processes to encourage women to return for follow-up and rescreening. To optimize access and follow-up of single-visit approach services, the appropriate cervical cancer prevention approach—whether to provide single-visit approach services at primary health centers versus secondary and tertiary facilities and the strategy and types of providers used for client follow-up—must also be carefully considered in each country setting.

The VIA follow-up results in this project indicate that the great majority (90%) of women who were initially tested as VIA+ were VIA- 1 year later. This very high potential cure rate of 90% seen in women rescreened 1 year later was also seen during the 2-year follow-up, in which all (100%) women who returned for screening were VIA-. The high potential cure rate shows the benefit of the single-visit approach and suggests that women with HIV in this population were not severely immunocompromised or were on ART.

Regional variation in treatment and follow-up rates with the single-visit approach can partially be attributed to the health facility’s ability to coordinate efforts across different stakeholders, developing systems to ensure success in implementing the single-visit approach, and variability in clinical competency. For example, health facilities in the region of Tigray were able to provide comprehensive client counseling in the local language; provide coordination between single-visit approach service providers, staff from the HIV/AIDS care unit, and PLHIV associations; and allocate resources for client follow-up and rescreening. Variability in the skills and competency of health care providers, either through differences in quality improvement measures or availability of specialized health care providers (i.e., gynecologists) within a health facility, could also result in regional variability of VIA+ diagnosis and treatment rates.

No major concerns were noted from single-visit approach beneficiaries; however, several women with HIV would ask why this service was being offered only to them. Due to resource constraints in the country and the fact that this was a pilot implemented in a few health facilitates, trained health care providers would carefully explain to those that were concerned that people with weakened immune systems, such as individuals with HIV, were at higher risk of cervical cancer compared with the general population and that the single-visit approach was currently offered only to women with HIV.

### Lessons Learned

The results of the HFA conducted as part of the *Addis Tesfa* project highlighted some of the health systems challenges that need to be considered and addressed to ensure successful cervical cancer prevention and control programs in resource-constrained settings:

- Private rooms with sufficient space and lighting are needed for counseling and delivery of the single-visit approach.
- Back-up systems for clean water supply must be devised accordingly.
- Cryotherapy machines must be fully functional with supplies and spare parts readily available.
- Coordination and forecasting systems for regular maintenance, back-up machines, and monitoring of essential supplies are important components that need to be integrated into the operational management of health facilities.
- The human resources necessary to implement the single-visit approach for cervical cancer prevention must also be appropriately managed. With health care provider turnover experienced in the majority of single-visit approach sites in Ethiopia, training multiple health care providers at each site, especially groups of nurses and midwives, was an important strategy employed by the project in ensuring consistent and sustainable single-visit approach services for cervical cancer prevention. To maximize the public health impact of cervical cancer prevention, rigorous training and support of health care providers must be an integral component of any single-visit approach program.

Training multiple health workers at each project site helped ensure consistent and sustainable services in face of high staff turnover.

### Addis Tesfa Experience and Future of Cervical Cancer Prevention in Ethiopia

The strong partnership between implementers, government representatives, and technical experts led to a collaborative endeavor that supported the first cervical cancer prevention program in Ethiopia to implement the single-visit approach. Despite health facility challenges described in some project-supported sites, the overall results of the project suggest that the single-visit approach for cervical cancer prevention services for women with HIV can be implemented in secondary and tertiary health facilities in resource-constrained settings.

Several important outcomes have resulted from the *Addis Tesfa* project. First, the project was able to work with the FMOH and key stakeholders to develop the “Guidelines for Cervical Cancer Prevention and Control in Ethiopia” that integrates the single-visit approach into the national reproductive health strategy. In addition, the project has expanded cervical cancer prevention services beyond women with HIV to include all eligible women who are in need of VIA screening. Despite the health system constraints, the project laid the groundwork and generated interest for phased scale-up of cervical cancer prevention services in Ethiopia, an initiative spearheaded by the first lady of Ethiopia, Roman Tesfaye Abneh, now working toward offering the single-visit approach in more than 100 facilities in 2015. As part of the national commitment to address cervical cancer and phased scale-up of single-visit approach services, a strong cervical cancer prevention network of implementers, government representatives, and technical experts must continue to monitor and provide technical guidance to the prevention strategy in Ethiopia. In addition, continued investments to increase availability and functionality of cryotherapy machines, while supporting ongoing training and capacity building of health care providers in key single-visit approach services (VIA, cryotherapy, and LEEP), are critical components of the national cervical cancer prevention agenda.

### Limitations

This study was designed to describe and learn about the implementation of the *Addis Tesfa* project using routine client data and health facility assessments. Performance monitoring data may identify statistically significant differences in single-visit approach results among the different regions; however, the study was not designed to explore and evaluate regional variability in great detail. In addition, client data were analyzed at the aggregate level and did not contain adequate individual-level variables, which prevented regression and correlation analysis of single-visit approach outcomes against client characteristics (i.e., ART status, CD4 count, etc.).

## CONCLUSIONS

The high rates of VIA screening and cryotherapy treatment in the *Addis Tesfa* project suggest that cervical cancer prevention services among women with HIV can be implemented in secondary- and tertiary-level health facilities using the single-visit approach in Ethiopia. However, success of prevention programming must also consider the health system challenges to ensure sustainability and appropriate scale-up of single-visit approach services in Ethiopia and other resource-constrained settings. To optimize access and follow-up of such services, the appropriate cervical cancer prevention approach—identifying the optimal types of facilities, service providers, and entry points for single-visit approach services—must be carefully considered in each country setting.

## Notes

**Peer Reviewed**
